# Can you escape rehab? Theoretical and design foundations of an online escape room for speech-language pathology students

**DOI:** 10.4102/sajp.v82i2.2285

**Published:** 2026-04-30

**Authors:** Nancy Barber

**Affiliations:** 1 Department of Speech Pathology and Audiology, Faculty of Humanities, University of the Witwatersrand, Johannesburg, South Africa

**Keywords:** educational technology, online escape room, clinical reasoning, speech-language pathology education, healthcare education, capacity building

## Abstract

**Background:**

Speech-language pathology (SLP) students in South Africa often face persistent challenges in transferring theoretical learning to authentic clinical contexts. This theory–practice divide is compounded by limited placement opportunities, multilingual classrooms, and broader structural inequities, all of which contribute to a significant theory–practice divide. Addressing these contextual realities requires innovative pedagogical approaches that extend beyond traditional teaching methods.

**Objectives:**

This article presents the conceptual and theoretical foundations of a low-cost, online escape room aimed at enhancing clinical reasoning skills in third-year SLP students, grounded in the contextual realities of South African higher education.

**Method:**

The design was informed by social constructivism, self-determination theory, and simulation-based learning. Implemented via the Genial.ly platform, the escape room integrated authentic case-based tasks, facilitator-mediated feedback, progress-dependent sequencing, and multimedia layering. These features created an interactive, psychologically safe space where students could apply theoretical knowledge to contextually relevant clinical challenges.

**Results:**

The design foregrounds social learning, motivation, and contextual responsiveness – core dimensions of capability building in resource-limited systems. It demonstrates how theoretically informed, technology-enhanced learning can promote autonomy, competence, and relatedness, supporting the development of confident and reflective practitioners in multilingual and under-resourced environments.

**Conclusion:**

This article proposes a scalable and contextually responsive model for developing clinical reasoning in the Global South healthcare education contexts. By foregrounding theoretical grounding and contextual adaptability, it contributes to the broader conversation on how technology-enhanced learning can support capability development in resource-limited settings.

**Clinical implications:**

The online escape room illustrates the potential of low-fidelity, technology-enhanced simulations to improve clinical reasoning, learner engagement, and confidence in diverse, resource-constrained training contexts, providing an equitable, scalable means to bridge theory and practice.

## Introduction

Escape rooms are increasingly being used in health professions education as an innovative tool to promote authentic clinical reasoning, collaboration, and learner engagement (Moore & Campbell 2021; Van Gaalen et al. 2021). Escape rooms are time-bound, team-based problem-solving activities requiring participants to solve puzzles to ‘escape’ a designated scenario. Effective educational escape rooms incorporate game mechanics (time pressure, narrative, collaboration), clear learning objectives, and immediate feedback (Davis et al. 2022; Shah, Pitt & Norton 2023). The gamified, team-based nature of escape rooms provides a refreshing alternative to traditional pedagogies, particularly when integrated with case-based learning (Fotaris & Mastoras 2019).

However, most applications of escape rooms have been developed in well-resourced contexts in the Global North, which broadly refers to high-income, industrialised nations in Europe, North America and parts of East Asia. Development of escape rooms for healthcare education in the Global North often overlooks the unique challenges and educational imperatives of institutions in the Global South, which comprises lower- and middle-income countries in regions such as sub-Saharan Africa, Latin America and parts of Asia. In South Africa, for instance, speech-language pathology (SLP) students must navigate the legacy of educational inequality, multilingualism, and limited clinical placement opportunities (Abrahams et al. 2019; Khoza-Shangase & Mophosho 2021; Pillay et al. 2020). These realities call for contextually responsive teaching strategies that do not simply replicate imported models but are grounded in local needs and constraints (Watermeyer & Neille 2022).

Considering these factors, healthcare professions’ education also seeks to bridge the theory–practice divide by engaging students in authentic, real-life experiences (McCallum et al. 2019). This approach helps students to better understand theoretical knowledge, enhances clinical reasoning, and builds professional self-confidence. Despite these benefits, students often struggle to apply theoretical knowledge to complex clinical cases, highlighting the challenge of integrating theory with practice (Benson, Provident & Szucs 2013; Hakim et al. 2014).

This article presents the theoretical rationale and design of an online escape room activity developed for third-year SLP students in a South African university. In doing so, it contributes a much-needed Global South perspective to the growing body of literature on escape rooms in health professions education. The following review of literature situates this innovation within current scholarship on escape rooms in health professions education and highlights the specific gaps this study seeks to address.

## Literature review

Recent scoping reviews of using escape rooms in healthcare education confirm that escape rooms can improve learner engagement, satisfaction, and teamwork while also supporting the acquisition of clinical reasoning skills (Nowbuth & Parmar 2024; Park et al. 2025). Park et al. (2025) highlight that escape rooms often foster specific competencies, particularly in communication, collaboration, and leadership. Their review also notes that most escape rooms are implemented in medicine and nursing, with limited application in allied health fields, including SLP. Nowbuth and Parmar (2024) similarly emphasise that escape rooms are underutilised in allied health education despite their adaptability.

Shah et al. (2023) provide practical guidelines for puzzle design and stress the importance of cognitive alignment, which ensures that the mental processes required to solve puzzles are congruent with the intended learning outcomes. In addition, Moffett et al. (2023) argue that escape rooms can provide insight into learners’ approaches to uncertainty management, a critical skill in healthcare. Their work demonstrates that escape rooms can be used not just for teaching content but for metacognitive development. Davis et al. (2022) offer 12 implementation tips, including clarity of objectives, facilitation roles, and thoughtful debriefing to enhance the educational impact.

However, the theoretical underpinnings of escape room pedagogy remain inconsistently applied. While many studies cite improved engagement, few link design elements to robust theories of learning. Additionally, the boundaries between simulations and escape rooms are often blurred. Unlike simulations that replicate real-life clinical encounters with fidelity, escape rooms introduce contrived challenges structured around game-based logic. Their pedagogical value lies in blending content review with puzzle-solving, often in a low-stakes, high-engagement format (Moffett et al. 2023).

Despite growing global interest, little attention has been paid to how escape rooms function in the resource-limited, culturally diverse educational contexts of the Global South. Most existing literature focuses on well-resourced, often monocultural institutions in the Global North, where escape rooms are implemented using sophisticated simulation labs and dedicated educational designers. In contrast, educators in the Global South must navigate large classes, digital inequities, and highly heterogeneous student cohorts (Timmis & Valladares 2025). Yet, these constraints also present opportunities for innovation. In many South African institutions, where the legacy of apartheid continues to shape unequal access to foundational education and technology (Morreira et al. 2020), educators must adopt low-cost, flexible approaches that support diverse learning needs. Multilingualism and multiculturalism are daily realities in these classrooms, necessitating tasks that are not only clinically relevant but also linguistically and culturally accessible (Khoza-Shangase & Mophosho 2021).

Much of the existing research on SLP education in South Africa, as with other allied healthcare education, highlights the need to reform academic pedagogies. This reform is required to ensure that clinical practice is equitable, just, and contextually relevant (Khoza-Shangase & Mophosho 2021). Researchers have raised concerns that graduate therapists may lack the necessary skills to deliver effective and relevant therapy to the communities they serve (Abrahams et al. 2019). This skills gap reflects a persistent theory–practice divide, whereby students struggle to transfer theoretical knowledge from the classroom into the complexities of clinical contexts (Mupawose, Adams & Moonsamy 2021; Rapillard, Plexico & Plumb 2019). While clinical environments demand patient-centred understanding, emotional intelligence, and practical skill application, academic programmes have often emphasised knowledge building with limited authentic engagement (Rapillard et al. 2019).

Educational innovations such as escape rooms can help to address this challenge of bridging the theory-practice divide by immersing students in interactive, problem-solving scenarios that simulate clinical complexity in a safe, structured environment (Nowbuth & Parmar 2024; Park et al. 2025). By requiring learners to interpret data, make decisions, and justify their reasoning in real time, escape rooms create opportunities to practise and integrate theoretical knowledge with practical application before entering actual clinical placements (Nowbuth & Parmar 2024; Park et al. 2025). Addressing this divide requires pedagogical strategies that intentionally bridge theory with practice, preparing students to navigate the demands of real-world healthcare delivery.

This article positions the online escape room as a pedagogical strategy, which reflects an intentional shift towards approaches that are context-sensitive, socially responsive, and grounded in the lived realities of Global South students. In the South African context, where clinical exposure and teaching resources may be constrained, escape rooms offer a scalable and engaging means of preparing students for real-world complexity. By foregrounding this context, this article contributes a critical, underrepresented voice to the global literature on escape room design in health professions education. Firstly, the conceptual framework of the escape room design will be presented. Secondly, the underlying pedagogies will be discussed to highlight the design of the escape room so that it is relevant to the South African context and the students’ needs.

## Conceptual framework of the escape room design

The escape room ‘Escaping Rehab: Complete all the missions to facilitate community reintegration’ was designed for third-year SLP students at the University of the Witwatersrand, which is a South African university, to support the application of theoretical knowledge in a simulated, authentic context.

The design was underpinned by social constructivism, self-determination theory, and simulation-based learning, and grounded in a socially responsive pedagogical framework that acknowledges the inequities shaping the Global South higher education (Tewari & Ilesanmi 2020; Vandeyar 2020). These theoretical perspectives collectively informed the structure, content, and learning processes embedded within the escape room.

Within a social constructivist paradigm, learning was understood as an interactive, situated process that occurs through collaboration and problem-solving (Thomas et al. 2014). The escape room therefore positioned students as active participants who co-constructed knowledge through joint reasoning and reflection. Tasks were designed to simulate authentic clinical encounters, requiring students to engage in diagnostic reasoning, goal setting, and decision-making aligned with real-world practice. The Genial.ly platform was chosen for its capacity to integrate multimodal and interactive design elements such as clickable hotspots, drag-and-drop puzzles, and conditional branching, allowing learners to engage with material in a sequenced yet non-linear way. These features created a safe space for learning that mirrored clinical decision-making, while providing structured scaffolding to consolidate understanding through feedback and reflection (Bearman et al. 2019).

During design, a variety of multimedia formats were incorporated, including written narratives, embedded videos, and scanned assessment forms, to build a rich, authentic clinical case. This deliberate use of multimedia was theoretically grounded in constructivist and experiential learning principles, which emphasise learning through sensory, emotional, and contextual engagement (Herrington, Reeves & Oliver 2010; Kolb 1984). The integration of diverse formats enabled learners to process information through multiple modalities, fostering deeper cognitive engagement and supporting the transfer of knowledge to practice (Moreno & Mayer 2007). This multimedia layering heightened realism and immersion, creating a more authentic representation of clinical complexity that mirrors real-world decision-making (Lateef 2010).

Guided by self-determination theory, the escape room and associated blended learning activities were intentionally designed to foster autonomy, competence, and relatedness, which are the three psychological needs central to sustaining motivation and developing capability (Cook & Artino 2016). Autonomy was facilitated through students having to select and apply the theoretical knowledge they had learnt in class for the collaborative problem-solving in the escape room. Competence was developed through structured, progressively complex challenges that required students to apply theoretical principles to authentic, contextually relevant scenarios as the ‘patient’ moved through the research process. Relatedness was cultivated through peer collaboration, dialogue, and mutual support as students jointly navigated the puzzles and reflected on their reasoning. These interlinked components are meant to collectively enhance student engagement and self-efficacy, which should, in turn, build confidence in their development of clinical reasoning for their context (Cook & Artino 2016).

The escape room’s design also reflected the principles of simulation-based learning, particularly its emphasis on experiential engagement, feedback, and debriefing (Bearman et al. 2019). As in other simulation modalities, students worked through a realistic patient case before participating in a facilitator-led debrief. This reflective phase aligned with the metacognitive cycle of simulation, where learners evaluated decision-making processes and linked theoretical constructs to practice-based reasoning (Rudolph et al. 2006). Through these structured interactions, the escape room served not simply as a ‘game’ but as a simulation, where a designed experience allowed theory to be enacted, explored, and hopefully internalised.

Importantly, the fictional case study within ‘Escaping Rehab’ was situated in the South African clinical context, tracing the patient’s trajectory across acute care, inpatient rehabilitation, outpatient management, and community reintegration. This localisation of content reflected the decolonial imperative to design learning activities that are culturally, linguistically, and socio-economically relevant to the context within which students learn and are expected to one day provide healthcare services (Vandeyar 2020). By embedding tasks in authentic local narratives and using accessible technology compatible with mobile devices and campus Wi-Fi, the design advanced both equity and contextual responsiveness in SLP education.

This commitment to contextual and socially responsive design also forms the foundation of the broader pedagogical orientation underpinning the escape room. The design principles that guided ‘Escaping Rehab’ extend beyond a single learning activity to reflect a wider response to the systemic and epistemic challenges facing SLP education in South Africa. The pedagogical framework described below situates this innovation within the realities of the Global South, foregrounding how context, equity, and authenticity inform the development of transformative and locally relevant learning experiences.

## Pedagogical foundations for contextually responsive learning in the Global South

The pedagogical framework for the escape room was developed in response to the structural and epistemic challenges characterising SLP education in South Africa and other Global South contexts. Students enter higher education from varied and often under-resourced schooling systems that may not have provided equitable preparation for university-level study. They also navigate multilingual environments and healthcare structures marked by historical and systemic inequities that continue to shape access to services (Burger & Christian 2020; Gordon, Jones & Goliath 2020). Within this reality, traditional pedagogical models imported from the Global North have tended to privilege theoretical mastery over contextually responsive application, leaving students underprepared for the complexity and diversity of South African clinical practice (Mupawose et al. 2021; Rapillard et al. 2019). Limited access to authentic learning opportunities during theoretical lectures further exacerbates this gap, resulting in graduates who struggle to integrate theory into culturally and linguistically appropriate care for patients in resource-constrained public healthcare settings (Abrahams et al. 2019; Khoza Shangase & Mophosho 2021).

**FIGURE 1:**
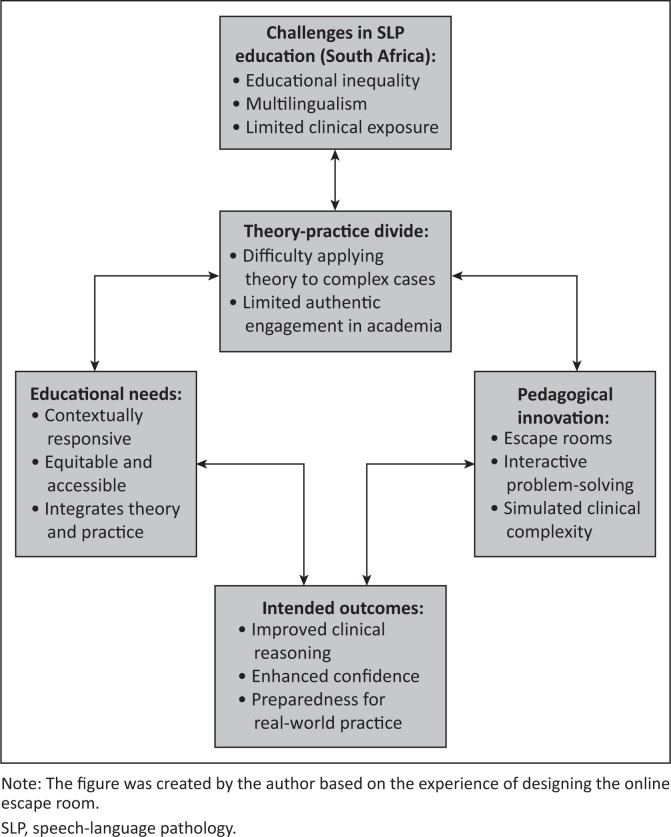
Pedagogical foundations for contextually responsive learning through the use of online escape rooms in speech-language pathology education.

Figure 1 illustrates the relationship between the contextual challenges, the theory–practice divide, the need for integrated learning approaches, and the role of escape rooms as a pedagogical innovation. This conceptual framework not only situates the escape room activity within broader initiatives to enhance SLP clinical education in South Africa but also informs the design of the intervention by aligning technological affordances with identified educational needs and intended learning outcomes.

Reimagining pedagogical design in such a context required methods that actively address inequities in access to experiential learning (Khoza Shangase & Mophosho 2021; Watermeyer & Neille 2022). The escape room operationalised this by providing a low-cost, high-impact simulation that allowed students to practise clinical reasoning safely before encountering patients in often high-pressure, under-resourced clinical environments. In South Africa, where placement sites can be unevenly distributed and patient loads are heavy, such digital simulations offer equitable exposure to realistic clinical scenarios. The use of multimedia case materials and interactive puzzles mirrored the continuum of patient recovery commonly observed in South African rehabilitation settings, enabling students to experience the interconnected stages of care across acute, inpatient, and community-based contexts. These pedagogical decisions were thus shaped by the need to design learning that is not only experiential but also attuned to the contextual realities of the South African healthcare system.

Collaboration and feedback were central to this framework because they reflect both social constructivist learning principles and the interdisciplinary nature of clinical practice in South Africa. In contexts where healthcare delivery depends on interprofessional collaboration and teamwork to overcome systemic shortages and resource limitations, developing these interpersonal and reflective skills early in training is crucial. Working in pairs enabled students to negotiate meaning, co-construct knowledge, and learn from diverse perspectives found in the multilingual and multicultural South African classroom (Coutts & Barber 2023). The embedded feedback mechanisms, both automated within the Genial.ly platform and facilitated through debriefing, encouraged reflection in action and reflection on action, skills necessary for continuous professional learning in resource-constrained and unpredictable clinical environments.

The conceptual framework thus positioned the escape room at the intersection of motivation, social learning, and contextual responsiveness. Through its integration of constructivist, self-determination, and simulation-based theories, the design offered a coherent, theory-informed response to the realities of SLP education in South Africa, where the balance between academic preparation and clinical exposure can be difficult to achieve. By fostering autonomy, competence, and relatedness (Cook & Artino 2016), the escape room aimed to support student motivation and confidence in navigating the complexities of healthcare service provision in the Global South. The online escape room was designed as a psychologically safe learning space where students could practise decision-making and bridge the theory–practice divide. The use of escape rooms in South African healthcare education remains in its early stages, yet their alignment with simulation pedagogy positions them as promising pedagogical innovations. Simulation is well-established as a means to support clinical decision-making, reduce anxiety before clinical placements, and enable the safe practice of complex skills (Bearman et al. 2019).

In under-resourced systems, where placement opportunities are limited and patient safety is paramount, low-fidelity, digital simulation variants offer a meaningful alternative. They also support academic lecturers and clinical educators who face increasing workloads, enabling structured practice without compromising patient safety. The online escape room therefore complements existing simulation approaches by offering a flexible, student-centred model of learning that can be integrated into curricula even in institutions with limited physical resources. Like other simulation-based methods, it also allowed students to rehearse clinical reasoning and decision-making before engaging with real patients, thereby mitigating risks and addressing constraints that contribute to the theory–practice divide.

The use of the Genial.ly platform was particularly suited to the Global South higher education context. Its web-based, mobile-compatible interface required no specialised hardware or costly licences, ensuring accessibility for students who rely on mobile devices and university Wi-Fi. In a country where digital inequity remains a barrier to participation, this choice promoted inclusivity and technological equity in higher education. The platform’s interactive design features, which include embedded feedback, multimedia layering, and progress-dependent sequencing, enabled a dynamic and responsive learning experience. These design features encouraged autonomy and critical thinking while enabling educators to scaffold learning in real time. Although formal learning analytics were not collected, educator reflections on student engagement patterns, such as time spent on tasks or repeated puzzle attempts, offered valuable insights into learner behaviour and areas of cognitive struggle. These insights reinforced the importance of design features that closely mimic the processes of clinical reasoning, enabling students to work through realistic case scenarios and strengthen their ability to connect theoretical principles with simulated practice. In future iterations, incorporating more robust data tracking could help monitor engagement, identify learning challenges, and refine task design for optimised adaptive scaffolding.

Looking ahead, online escape rooms hold potential to shape the future learning experience in health professions education in South Africa. With ongoing advancements in educational technology, these tools could evolve to incorporate adaptive feedback mechanisms or artificial intelligence to personalise learning pathways and adjust challenge levels in real time. Beyond SLP, the escape room model could be applied across health professions education for both medical and allied healthcare education. It could also be used in multiple stages of training to support the development of clinical reasoning, collaboration, and decision-making skills. In resource-constrained settings such as South Africa and the broader Global South, such innovations offer a scalable, contextually relevant approach to enhancing clinical education in health professions education. Most importantly, they provide a structured, engaging, and theory-informed means to bridge the theory–practice divide, which prepares students to navigate complex clinical realities but deliver equitable and culturally responsive healthcare.

## Conclusion

This article detailed the theoretical and contextual rationale for designing an online escape room tailored to third-year SLP students in a South African university. In addressing an identified gap in the literature, the study contributes a Global South perspective to the use of escape rooms in health professions education. The intervention offers an innovative, equitable, and contextually relevant approach to supporting clinical reasoning development in under-resourced, multilingual learning environments. Importantly, the online escape room was designed to help address the persistent theory–practice divide in SLP education by creating structured, authentic opportunities for students to apply theoretical knowledge in simulated clinical scenarios before engaging with real patients. While the project did not assess learning outcomes directly, it established a robust design foundation grounded in theory and local relevance. Future research should incorporate outcome measures, explore interprofessional adaptations, and further interrogate the pedagogical boundaries between simulations and game-based learning. In particular, integrating learning analytics and platform-based data tracking could enhance the evaluation of student reasoning processes, optimise task design, and ensure adaptive scaffolding in future digital learning environments in diverse global contexts.

## References

[CIT0001] Abrahams, K., Kathard, H., Harty, M. & Pillay, M., 2019, ‘Inequity and the professionalisation of speech-language pathology’, *Professions & Professionalism* 9(3), e3285. 10.7577/pp.3285

[CIT0002] Bearman, M., Greenhill, J., Nestel, D. & Rudd, C., 2019, ‘Simulation in health professional education: A sociomaterial view’, *Medical Education* 53(2), 64–74.30289171

[CIT0003] Benson, J.D., Provident, I. & Szucs, K.A., 2013, ‘An experiential learning lab embedded in a didactic course: Outcomes from a pediatric intervention course’, *Occupational Therapy in Health Care* 27(1), 46–57. 10.3109/07380577.2012.756599 23855537

[CIT0004] Burger, R. & Christian, C., 2020, ‘Access to health care in post apartheid South Africa: Availability, affordability, acceptability’, *Health Economics, Policy and Law* 15(1), 43–55. 29996951 10.1017/S1744133118000300

[CIT0005] Cook, D.A. & Artino, A.R., 2016, ‘Motivation to learn: An overview of contemporary theories’, *Medical Education* 50(10), 997–1014. 27628718 10.1111/medu.13074 PMC5113774

[CIT0006] Coutts, K. & Barber, N., 2023, ‘Peer learning model in speech-language pathology student practicals in South Africa: A pilot study’, *African Journal of Health Professions Education* 15(2), 2–6.

[CIT0007] Davis, K., Lo, H.Y., Lichliter, R., Wallin, K., Elegores, G., Jacobson, S. et al., 2022, ‘Twelve tips for creating an escape room activity for medical education’, *Medical Teacher* 44(4), 366–371. 33872114 10.1080/0142159X.2021.1909715

[CIT0008] Fotaris, P. & Mastoras, T., 2019, ‘Escape rooms for learning: A systematic review’, in L. Elbaek, G. Majgaard, A. Valente & S. Khalid (eds.), Proceedings of the 13th European conference on game based learning, ECGBL 2019, October 3–4, 2019, pp. 235–243, Academic Conferences and Publishing International Limited, Denmark.

[CIT0009] Gordon, N., Jones, C. & Goliath, V., 2020, ‘Language and access in higher education: A South African case study’, *Education as Change* 24(1), 1–20.

[CIT0010] Hakim, E.W., Moffat, M., Becker, E., Bell, K.A., Manal, T.J., Schmitt, L.A. et al., 2014, ‘Application of educational theory and evidence in support of an integrated model of clinical education’, *Journal of Physical Therapy Education* 28, 13–21. 10.1097/00001416-201400001-00005

[CIT0011] Herrington, J., Reeves, T.C. & Oliver, R., 2010, *A guide to authentic e learning*, Routledge, New York.

[CIT0012] Khoza-Shangase, K. & Mophosho, M., 2021, ‘Language and culture in speech-language and hearing professions in South Africa: Re-imagining practice’, *South African Journal of Communication Disorders* 68(1), a793. 10.4102/sajcd.v68i1.793 PMC8252163 34082547

[CIT0013] Kolb, D.A., 1984, *Experiential learning: Experience as the source of learning and development*, Prentice Hall, Englewood Cliffs.

[CIT0014] Lateef, F., 2010, ‘Simulation based learning: Just like the real thing’, *Journal of Emergencies, Trauma and Shock* 3(4), 348–352. 21063557 10.4103/0974-2700.70743 PMC2966567

[CIT0015] McCallum, C., Bayliss, J., Becker, E., Nixon-Cave, K., Colgrove, Y., Kucharski-Howard, J. et al., 2019, ‘The integrated clinical education strategic initiatives project – Development of parameters to guide harmonisation in clinical education: A scoping review’, *Physical Therapy* 99(2), 147–172. 10.1093/ptj/pzy135 30561697

[CIT0016] Moffett, J., Cassidy, D., Collins, N., Illing, J., De Carvalho Filho, M.A. & Bok, H., 2023, ‘Exploring medical students’ learning around uncertainty management using a digital educational escape room: A design-based research approach’, *Perspectives on Medical Education* 12(1), 86. 36969324 10.5334/pme.844 PMC10038110

[CIT0017] Moore, L. & Campbell, N., 2021, ‘Effectiveness of an escape room for undergraduate interprofessional learning: A mixed methods single group pre-post evaluation’, *BMC Medical Education* 21(1), 1–8. 33879150 10.1186/s12909-021-02666-z PMC8056636

[CIT0018] Moreno, R. & Mayer, R.E., 2007, ‘Interactive multimodal learning environments’, *Educational Psychology Review* 19(3), 309–326.

[CIT0019] Morreira, S., Luckett, K., Kumalo, S.H. & Ramgotra, M., 2020, ‘Confronting the complexities of decolonising curricula and pedagogy in higher education’, *Third World Thematics: A TWQ Journal* 5(1–2), 1–18. 10.1080/23802014.2020.1798278

[CIT0020] Mupawose, A., Adams, S. & Moonsamy, S., 2021, ‘Facilitators of and barriers to clinical supervision of speech-language pathology students in South Africa: A pilot study’, *African Journal of Health Professions Education* 13(1), 23–28. 10.7196/AJHPE.2021.v13i1.1216

[CIT0021] Nowbuth, A.A. & Parmar, V.S., 2024, ‘Escaping the ordinary: A review of escape rooms in medical and veterinary education’, *BMC Medical Education* 24(1), 1506. 39707331 10.1186/s12909-024-06512-w PMC11660942

[CIT0022] Park, G.L., Hegazy, S.A., Sepe, J., Swigart, J., Burnette, M., Beltran, J. et al., 2025, ‘Fostering competencies: A scoping review of escape rooms in Medical Education’, *Medical Science Educator* 35(2), 1–11. 40352986 10.1007/s40670-024-02270-y PMC12058590

[CIT0023] Pillay, M., Tiwari, R., Kathard, H. & Chikte, U., 2020, ‘Sustainable workforce: South African audiologists and speech therapists’, *Human Resources for Health* 18, 1–13. 10.1186/s12960-020-00488-6 32611357 PMC7329495

[CIT0024] Rapillard, S., Plexico, L. & Plumb, A.M., 2019, ‘Influence of supervision and clinical experience on professional development of graduate speech-language pathology students’, *Teaching & Learning in Communication Science & Disorders* 3(1), 1–28.

[CIT0025] Rudolph, J.W., Simon, R., Rivard, P., Dufresne, R.L. & Raemer, D.B., 2006, ‘Debriefing with good judgment: Combining rigorous feedback with genuine inquiry’, *Anesthesiology Clinics* 24(2), 361–376. 10.1016/j.anclin.2007.03.007 17574196

[CIT0026] Shah, A.S., Pitt, M. & Norton, L., 2023, ‘ESCAPE the boring lecture: Tips and tricks on building puzzles for medical education escape rooms’, *Journal of Medical Education and Curricular Development* 10, 23821205231211200. 38025020 10.1177/23821205231211200 PMC10664428

[CIT0027] Tewari, D.D. & Ilesanmi, K.D., 2020, ‘Teaching and learning interaction in South Africa’s higher education: Some weak links’, *Cogent Social Sciences* 6(1), 1740519. 10.1080/23311886.2020.1740519

[CIT0028] Thomas, A., Menon, A., Boruff, J., Rodriguez, A.M. & Ahmed, S., 2014, ‘Applications of social constructivist learning theories in knowledge translation for healthcare professionals: A scoping review’, *Implementation Science* 9(1), 1–20. 24885925 10.1186/1748-5908-9-54 PMC4040365

[CIT0029] Timmis, S. & Valladares-Celis, M.C., 2025, ‘Digital inequalities and the COVID legacy in higher education in the global South and North: Intersecting inaccessibilities and institutional assumptions’, *Compare: A Journal of Comparative and International Education* 1–19. 10.1080/03057925.2025.2483691

[CIT0030] Vandeyar, S., 2020, ‘Why decolonising the South African university curriculum will fail’, *Teaching in Higher Education* 25(7), 783–796. 10.1080/13562517.2019.1592149

[CIT0031] Van Gaalen, A.E., Brouwer, J., Schönrock-Adema, J., Bouwkamp-Timmer, T., Jaarsma, A.D.C. & Georgiadis, J.R., 2021, ‘Gamification of health professions education: A systematic review’, *Advances in Health Sciences Education* 26(2), 683–711. 33128662 10.1007/s10459-020-10000-3 PMC8041684

[CIT0032] Watermeyer, J. & Neille, J., 2022, ‘The application of qualitative approaches in a post-colonial context in speech-language pathology: A call for transformation’, *International Journal of Speech-Language Pathology* 24(5), 494–503. 10.1080/17549507.2022.2047783 35435778

